# Unpacking trust: The Italian validation of the Epistemic Trust, Mistrust, and Credulity Questionnaire (ETMCQ)

**DOI:** 10.1371/journal.pone.0280328

**Published:** 2023-01-26

**Authors:** Marianna Liotti, Alberto Milesi, Grazia Fernanda Spitoni, Annalisa Tanzilli, Anna Maria Speranza, Laura Parolin, Chloe Campbell, Peter Fonagy, Vittorio Lingiardi, Guido Giovanardi

**Affiliations:** 1 Department of Dynamic and Clinical Psychology, and Health Studies, Sapienza University of Rome, Rome, Lazio, Italy; 2 Department of Psychology, University of Milano-Bicocca, Milan, Lombardia, Italy; 3 Research Department of Clinical, Educational and Health Psychology, University College London, London, England, United Kingdom; Medical University of Vienna, AUSTRIA

## Abstract

The construct of epistemic trust has received much consideration in recent psychological literature, even though mainly from a theoretical perspective. The overall aim of this study was to validate the first self-report measure of epistemic trust–the Epistemic Trust, Mistrust, and Credulity Questionnaire (ETMCQ)–in an Italian sample. Our primary goal was to test the factorial validity of the instrument, also exploring the influence of age, gender, and level of education on epistemic trust (Study 1, n = 843). Secondarily, we investigated the associations between epistemic trust, mistrust, credulity, and other aspects of psychological functioning, as well as with the presence of adverse childhood experiences in a smaller number of participants (Study 2, n = 445). Besides the ETMCQ, the survey included an ad hoc questionnaire investigating socio-demographic characteristics and self-report measures of reflective functioning, mentalized affectivity, traumatic experiences, attachment, and psychological symptoms. Statistical analysis showed a three-factor hierarchical structure similar to the model proposed in the original validation, with some differences that suggest an influence of cultural factors in determining individuals’ epistemic stance. Our results corroborate previous theoretical contributions regarding the association between epistemic trust and psychological wellbeing, and between epistemic disruptions and higher levels of psychological suffering. Both Mistrust and Credulity were significantly related to the presence of childhood traumatic experiences, attachment avoidance and anxiety, lower levels of mentalization, lower abilities in emotional regulation, and higher levels of psychopathological symptoms. The ETMCQ represents an easily administered and time-effective tool. Its use could pave the way for interesting clinical and theoretical findings.

## Introduction

Epistemic trust (ET), defined as “the capacity of the individual to consider knowledge conveyed by others as significant, relevant to the self, and generalizable to other contexts” [1], has recently received attention for its theoretical and empirical relevance [1–7]. The construct of ET draws its origins from different disciplines, such as epistemology [8], philosophy [9], and sociology [10, 11], and potentially contributes to a better understanding of individuals’ relational life from several perspectives: developmental, social, pedagogical, and clinical. Recently, it has been proposed that ET plays a fundamental role as a protective factor against the development and maintenance of psychopathology [12, 13].

Building on Csibra and Gergely’s [14] natural pedagogy model and Sperber and colleagues’ [15] concept of epistemic vigilance, Fonagy and Allison [3] proposed a theoretical model in which ET is central to explain how humans transmit and acquire social and relational knowledge, linking this construct to the intergenerational transmission of attachment [16]. Caregiver-infant interpersonal exchanges are characterized by the presence of specific signals, called ostensive cues (e.g., turn-taking, eye contact, special vocal tone) that allow the communicator (the caregiver) to indicate that the upcoming message is trustworthy and personally relevant to the addressee (the infant). In the presence of secure attachment relationships––characterized by a sense of safety for the baby and by sensitive responses by the caregiver––this unique form of communication enables the child to lower their level of epistemic vigilance, opening the channel of ET and becoming able to accept the incoming information. Moreover, the communication strategies that characterize the caregiver-infant interpersonal exchanges, working as a special version of ostensive cues (such as marked mirroring; [17]), enable children to perceive the caregiver as a distinct being and themselves as an active agent in the relationship, thus acquiring the capacity for mentalizing [3].

Luyten, Campbell, and Fonagy [13] recently proposed a developmental model in which complex trauma (i.e., physical and/or emotional abuse or neglect within primary attachment relationships), in conjunction with neurobiological and psychological factors, may influence the individual’s development of ET, already interwoven with attachment quality and mentalization abilities. In some cases, dysfunctions in epistemic stance caused by early adversity may lead the individual to adopt a rigid and pervasive hypervigilant position toward information coming from others, resulting in high levels of Epistemic Mistrust (EM). This state of epistemic mistrustful petrification may heighten the risk of developing psychopathology [12, 13] perhaps via the mediation of social isolation and loneliness [18]. In other instances, adverse experiences may disrupt the ability to discriminate the information received from others, leading to epistemic credulity (EC)––that is, excessive and inappropriate trust in others, making the individual vulnerable to manipulation or mistreatment [19]. Both EM and EC might interfere with the ability to adaptively respond to interpersonal difficulties, hindering psychotherapy effectiveness. On the contrary, Fonagy and colleagues [19] have proposed that good levels of ET are vital in successful psychotherapy interventions. The most relevant element of innovation in this model is the focus on how individual differences regarding epistemic trust might develop, and on what could be the consequences and associations of different forms of epistemic disruptions.

This, however, has remained mainly a theoretical concept. Although valuable experimental procedures have already been developed to assess ET [20, 21], these are quite burdensome measures that do not allow a time-effective evaluation. Recently, Campbell and colleagues [1] have provided a vital contribution: the development and validation of the Epistemic Trust, Mistrust, and Credulity Questionnaire (ETMCQ). The ETMCQ represents the first self-report measure of epistemic trust and its dimensions, and it could prove to be extremely valuable both in clinical and research contexts. The authors proposed a 15 items self-report measure to assess various forms of epistemic stance, building on Grice’s [22] theory and relevance theory [23, 24]. Results provided evidence for a three-factor structure of the instrument, both in exploratory and confirmatory analyses, empirically substantiating the hypothesis of three separate epistemic stances. The questionnaire also showed good reliability and validity [1]. Significant associations were found between both EM and EC and low mentalizing abilities, as well as higher levels of childhood adversity, insecure attachment, and symptoms of mental health disorder. In addition, EM and EC were found to partially mediate the relationship between early adversities and psychopathology.

From these considerations, it is possible to underline the contribution that the construct of ET brings in our comprehension of how cultural and relational knowledge is transmitted, and the relevance of its operationalization from a theoretical, clinical, and empirical point of view. Adopting an evolutionary perspective, this model may help us understand how individuals acquire and share information relevant to their survival and adaptation, and how individual differences in this acquisition and sharing processes are created [1, 3, 25, 26]. Moreover, as already noted, restoring adequate levels of ET may be at the heart of all effective psychotherapeutic interventions [19, 27]. Research about the empirical validity of this construct and its measurement is therefore needed. Furthermore, its strong cultural component raises the question of the effect that cultural factors may have on the development of this psychological dimension, which requires investigation.

The overall aim of the present research was to validate the ETMCQ in a cohort of Italian adults. More specifically, we aimed to 1) test the factorial structure of the instrument (via Principal Component Analysis and Confirmatory Factor Analysis); 2) test the mean differences in ET, EM and EC levels between males and females (as done by Campbell and colleagues [1] as well as the correlations between the three different epistemic stances and sociodemographic characteristics such age and educational level; and 3) investigate the associations between ET, EM, and EC and other aspects of psychological functioning, as well as with the presence of adverse childhood experiences.

## Methods

### Procedure and participants

Before its administration, ETMCQ has been translated into Italian by the authors and subsequently back-translated into English by an independent translator. To verify the accuracy of the first translation, the two versions were compared through qualitative analysis, and no significant differences emerged. The study was then carried out through an online survey using the platform Qualtrics. Participants were recruited through snowball sampling–i.e., via mailing lists and social media channels (e.g., diffusing the survey across popular Facebook pages and Instagram profiles, which proved to be extremely valuable for recruitment). The study consisted of two parts: first, we asked participants to complete an ad-hoc questionnaire designed to collect socio-demographic data (age, gender, level of education, and profession) and the ETMCQ (Study 1). Then we asked them to contribute to the second part of the study, which involved responding to additional self-report measures aimed at investigating the relationship between ETMCQ scores and other psychological dimensions such as reflective functioning, mentalized affectivity, adverse childhood experiences, attachment, and psychopathology symptoms (Study 2).

Eight hundred and forty-three participants responded to the ad hoc-questionnaire and the ETMCQ (Study 1, n = 843), while four hundred and forty-five also responded to the questions contained in the second form (Study 2, n = 445). Socio-demographic features of the global sample (Study 1, n = 843) were as follows: 425 subjects were females (50.4%), 417 males (49.5%), and 1 (0.1%) non-binary. The age range was from 18 to 80 years old (M = 32.47, SD = 12.88). All subjects were Italian. Educational levels were as follows: 188 subjects (22.4%) completed only high school, 303 (35.9%) had a bachelor’s degree, 302 (35.8%) had a master’s degree, and 50 (5.9%) had completed a Ph.D. Socio-economic status was very low (i.e., 0–10.000 euros per year) for 11 subjects (1.3%), low (i.e., 10.000–25.000 euros per year) for 268 (31.8%), medium (i.e., 25.000–45.000 euros per year) for 408 subjects (48.4%), and high (i.e., 45.000 euros or more per year) for 156 (18.3%). Regarding marital status, 267 subjects (31.4%) were single, 384 (45.2%) married or in a stable relationship, 182 (21.4%) separated or divorced, 7 (0.8%) widowed, and 10 (11.8%) preferred not to specify it.

In each case, the IP address was logged to prevent duplicate answers. We granted complete anonymity to all responders. Subjects could decide to interrupt the survey at any time and resume it afterward or revoke their participation entirely. The survey has been online from June 2021 to March 2022. Inclusion criteria were being aged 18 years or older and speaking Italian. No participants were excluded, and all of them gave written informed consent to participate in each study. Respondents were not compensated in any way. The study was approved by the ethics review board of Sapienza University of Rome (Protocol N. 0001292). All procedures performed in this study were in accordance with the Helsinki declaration standards and its later amendments.

### Additional measures (Study 2)

**The Reflective Functioning Questionnaire** (RFQ; [28]), an eight-item self-report instrument to evaluate mentalization. It assesses the ability to understand one’s own and others’ behaviors in terms of underlying mental states (e.g., intentions, points of view, values, desires, and emotions). It asks subjects to express their agreement with each item on a seven-point Likert scale (ranging from 1 = “completely disagree” to 7 = “completely agree”). Although the scoring of the original instrument evaluated two dimensions: *certainty* (RFQ_C) and *uncertainty* (RFQ_U) in relation to mental states, Woźniak-Prus and colleagues [29] recently found more robust results using a single dimensional structure that assesses uncertainty about mental states (hypomentalization). Higher levels of uncertainty regarding mental states were found to be correlated with difficulties in affect regulation, symptoms of psychopathology (e.g., borderline personality features, depressive and anxiety symptoms), interpersonal problems, and attachment anxiety. The Italian validation of the RFQ shows good internal consistency, construct validity, and test-retest reliability [30].

**Brief-Mentalized Affectivity Scale** (B-MAS; [31]), a 12-item self-report instrument developed to evaluate mentalized affectivity, a construct that combines several previous contributions from mentalization theory and emotional regulation models. Mentalized affectivity theory [32] postulates that our affective regulation abilities depend upon our capacity to reflect on mental states and previous experiences through autobiographical memory. The instrument assesses three dimensions: the ability to *identify* emotions (e.g., to be aware of their presence and accurately label them), *process* them (e.g., to modulate their intensity), and *express* them (e.g., to communicate feelings and their meaning). The B-MAS has recently been validated in Italian [33], showing excellent psychometric properties, good reliability, and factorial and convergent validity.

**Childhood Trauma Questionnaire–Short Form** (CTQ-SF; [34]), a 28-item self-report instrument developed to assess retrospectively adverse childhood experiences such as abuse and neglect. For each item, subjects must rate how often they suffered a specific adverse experience using a five-point Likert scale (ranging from 1 = “never true” to 5 = “very often true”). The instrument evaluates the presence and severity of five types of traumatic childhood experiences (Physical Abuse, Emotional Abuse, Sexual Abuse, Emotional Neglect, and Physical Neglect). High scores at the CTQ–SF correlate with depressive, dissociative, and post-traumatic stress disorder symptoms [35]. The Italian validation of the instrument has confirmed a five-factor model with good reliability, structure, and concurrent validity [36].

**Experiences in Close Relationships-Revised** (ECR-R; [37]), a self-report tool consisting of 18 items and investigating adult romantic attachment styles. Subjects are asked to refer to how they generally behave in relationships, and to rate their level of agreement with each item using a seven-point Likert scale (ranging from 1 = “strongly disagree” to 7 = “strongly agree”). The instrument evaluates two main dimensions: *attachment-related anxiety* (intense concern for relationships, fear of abandonment) and *attachment-related avoidance* (feelings of discomfort in establishing emotional closeness with the partner, difficulty in trusting others). The Italian validation shows good test-retest reliability and internal consistency [38].

**Brief Symptoms Inventory** (BSI; [39]), a 53-item self-report instrument investigating nine dimensions of psychopathological symptoms: somatization, obsession-compulsion, interpersonal sensitivity, depression, anxiety, hostility, phobic anxiety, paranoid ideation, and psychoticism. The tool also allows the evaluation of three general indices of distress: global severity index, positive symptom distress index, and positive symptom total. For each item, subjects must indicate how often they experienced a particular symptom in the last week using a five-point Likert scale (ranging from 0 = “not at all” to 4 = “extremely”).

### Data analysis

Statistical analyses were performed using IBM SPSS Statistics 27.0. Jamovi project software (Version 1.8.1) was used for Principal Component Analysis (PCA) and Confirmatory Factor Analysis (CFA). Before running statistical tests, all data were examined for normality (skewness and kurtosis). The criterion for significance was set at *p* = 0.05 for all analyses.

In addition to descriptive statistics, we evaluated the following psychometric properties of the ETMCQ:

1. **PCA**. A Principal Component Analysis (PCA with Varimax rotation) was performed to reduce the dimensionality of the scale. The number of factors was chosen according to experts’ recommendations (Kaiser-Meyer-Olkin criteria > 0.6, Bartlett’s Test of Sphericity <0.05, and parallel analyses). These indices were used to explore the simple structure.
2. **CFA**. Confirmatory Factor Analysis (CFA) was run to test the emerged structure. Standardized coefficients of ≥ 0.4 were considered acceptable (Brown, 2006). Normalized mean and covariance residuals were evaluated and found acceptable. Maximum Likelihood (ML) was used as an estimation method.
3. **Model fit** was estimated by two absolute indices of overall model fit: root mean square error of approximation (RMSEA) and standardized root mean residual (SRMR). Additionally, one relative index of model fit was used: comparative fit index (CFI). The acceptable thresholds for these indices were defined as RMSEA  =  0.05–0.08, SRMR < 0.08, and CFI > 0.90, according to Kline’s guidelines [40].
4. **Cronbach’s alpha** was calculated for having an estimate of the internal consistency of the scale.
5. **Independent sample t-test** was run to investigate the mean difference of each dimension between males and females (divided by assigned at birth gender).
6. **Pearson’s correlation coefficient** was calculated to investigate the relationship between epistemic dimensions and age and other variables object of the study.
7. **Spearman’s correlation coefficient** was calculated to explore the association between epistemic dimensions and level of education.

## Results

### Study 1

PCA and CFA were conducted on independent samples. The original sample was divided into two halves: PCA was conducted on the first half, while CFA was studied on the remaining half. The division was done by assigning a random number to each subject in the study (excel rand function) and dividing the sample into even numbers (50%) and odd numbers (50%). The two halves were then named “discovery sample” (even numbers) and “confirmation sample” (odd numbers).

**Fig 1:**
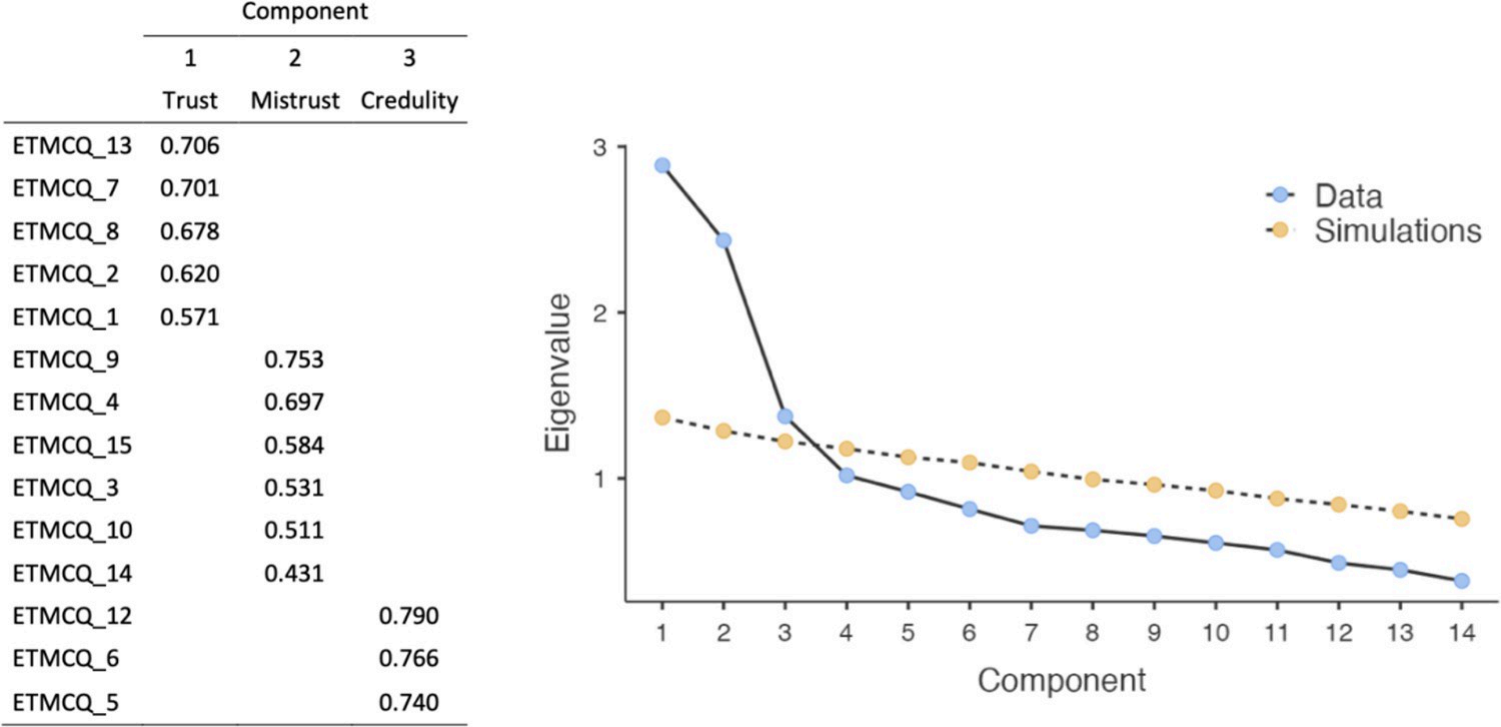
On the left: Loadings of the three factors after Varimax rotation. Due to a software property only loads >0.3 are reported. On the right: Scree-test and parallel analysis plot.

The PCA on the original scale, consisting of 15 items, showed a three-factor structure with an explained variance of 48.7%. In this first solution, item 11 showed similar and low saturations on components 2 and 3 (0.416 and 0.401, respectively), suggesting a difficult allocation of the item in the structure. To make the solution clearer, item 11 was removed from the analysis, and a subsequent PCA was conducted on the remaining 14 items. The rotated factor pattern (Fig 1 panel A) revealed a simple structure consisting of three components; the explained variance was 47.8%. The Kaiser-Meyer-Olkin Measure of Sampling Adequacy was .743, and Bartlett’s Test of Sphericity was significant (*X*^2^ = 1110; *p* <0.01).

As shown in Fig 1, all variables present high loadings on only one component and very low loadings on all the others. Moreover, there were no correlations (Pearson r) among the three components suggesting independence among all of them. In Table 1 SS loadings and variance of the components are shown.

**Table 1:** SS Loadings and variances of the components.

Component	SS Loadings	% of Variance	Cumulative %
1	2.32	16.6	16.6
2	2.31	16.5	33.1
3	2.07	14.8	47.8

We named the components with the same labels used in the original work by Campbell and colleagues [1], namely: Trust (component 1; items 1, 2, 7, 8, 13), Mistrust (component 2; items 3, 4, 9, 10, 14, 15), and Credulity (component 3; items 5, 6, 12).

The CFA provided a statistically significant model (χ^2^ = 245; p < 0.001). As shown in Table 2, the fit statistics provided acceptable values.

**Table 2:** Fit measures.

			RMSEA 90% CI	
CFI	SRMR	RMSEA	Lower	Upper
.90	.064	.074	.064	.084

Note: CFI = comparative fit index; SRMR = standardized root mean square residual; RMSEA = root mean square error of approximation.

Finally, the internal consistency of each of the three scales, analyzed with Cronbach’s alpha, showed acceptable values (see Table 3).

**Table 3:** Scale reliability statistics.

	Cronbach’s α
Trust (component 1)	0.728
Mistrust (component 2)	0.672
Credulity (component 3)	0.728

#### Correlations with demographic variables

ET means in the male group (M = 4.86; SD = 1.07) did not differ significantly from ET means in the female group (M = 4.99; SD = 1.01), t(840) = -1.85, *p* = .252. EM means in males (M = 3.57; SD = 1.03) did not differ significantly from EM means in females (M = 3.72; SD = 1.06), t(840) = -2.17, *p* = .638. EC means in the male group (M = 2.28; SD = 1.17) did not differ significantly from EC means in the female group (M = 2.42; SD = 1.27), t(840) = -1.68, p = .174.

Age was negatively associated only with EM, r(843) = -.16, *p*< .001. It showed no association with ET, r(843) = .01, *p* = .818, nor EC, r (843) = -.03, *p* = .385. Similarly, level of education was negatively associated only with EM, r(843) = .13, *p* < 0.001. No association was found with ET, r(843) = 0.06, *p* = .094, nor with EC, r(843) = -.019, *p =* .572.

### Study 2

#### Convergent validity

Means, standard deviations, and zero-order correlations for each variable included in the study are shown in Tables 4 and 5.

**Table 4:** Mean (M) and Standard-Deviations (SD) of the variables studied.

	M	SD
ET	4.73	1.05
EM	3.65	1.05
EC	2.47	0.98
Hypo-M	3.51	0.96
Child-Trauma	36.45	10.89
Att-Avoid	4.06	0.57
Att-Anx	3.84	0.51
GSI	0.27	0.19
MA-Id	5.47	1.22
MA-Elab	4.21	1.35
MA-Expr	3.69	1.44

Note: Epistemic Trust (ET), Epistemic Mistrust (EM), Epistemic Credulity (EC), hypomentalization (Hypo-M), Childhood Traumatic Experiences (Child-Trauma), Attachment Avoidance (Att-Avoid), Attachment Anxiety (Att-Anx), psychological symptomatology (GSI), Mentalized Affectivity Identification (MA-Id), Mentalized Affectivity Elaboration (MA-Elab), Mentalized Affectivity Expression (MA-Expr).

**Table 5:** Correlations between the main variables of the study.

	ET	EM	EC
RF (Hypo-M)	.04	.44**	.21**
Child-Trauma	-.15**	.39**	.11*
Att-Avoid	-.19**	.37**	.21**
Att-Anx	-.03	.50**	.10**
GSI	-.10*	.55**	.11**
MA-Id	.23**	-.01	-.06
MA-Elab	.08	-.39**	-.13**
MA-Expr	.36**	-.36**	.39**

Note: Epistemic Trust (ET), Epistemic Mistrust (EM), Epistemic Credulity (EC), hypomentalization (Hypo-M), Childhood Traumatic Experiences (Child-Trauma), Attachment Avoidance (Att-Avoid), Attachment Anxiety (Att-Anx), psychological symptomatology (GSI), Mentalized Affectivity Identification (MA-Id), Mentalized Affectivity Elaboration (MA-Elab), Mentalized Affectivity Expression (MA-Expr).

** p < .001

* < .05.

Several positive and negative associations were found between the three epistemic stances (ET, EM, and EC) and the other variables investigated. Specifically, ET was positively associated with the Mentalized Affectivity Identification (MA-Id) dimension and the Mentalized Affectivity Expression dimension (MA-Expr), and negatively associated with childhood traumatic experiences (Child-Trauma), Attachment Avoidance (Att-Avoid), and the general degree of severity of mental health symptomatology (GSI). EM was positively associated with hypomentalization (Hypo), childhood traumatic experiences (Child-Trauma), Attachment Anxiety (Att-Anx), Attachment Avoidance (Att-Avoid), and the general degree of severity of psychological symptomatology (GSI); it was also negatively associated with Mentalized Affectivity Elaboration dimension (MA-Elab) and Mentalized Affectivity Expression dimension (MA-Expr). Last, EC was positively associated with hypomentalization (Hypo), childhood traumatic experiences (Child-Trauma), Attachment Anxiety (Att-Anx), Attachment Avoidance (Att-Avoid), and the general degree of severity of mental health symptomatology (GSI), and negatively associated with the Mentalized Affectivity Elaboration dimension (MA-Elab).

## Discussion

To validate the ETMCQ in the Italian sample (the overall aim of the present study), our primary goal was to evaluate the factorial structure of the questionnaire. Findings from the PCA and CFA mirrored the model proposed by Campbell and colleagues [1], revealing a three-factor structure. As in the original English version, the first extracted component was Trust, measuring people’s ability to consider new knowledge coming from others as truthful and relevant; the second one was Mistrust, measuring people’s tendency to consider the information coming from others as untrustworthy (if not potentially damaging), suggesting wariness in relation to social learning; the third one was Credulity, measuring the inclination to overly rely on information coming from others, lacking epistemic vigilance and thus being susceptible to deception or manipulation within social relationships. Our results support previous contributions [1, 15, 19, 41] regarding the presence of at least three epistemic strategies: one functional and two suggesting epistemic dysfunction.

Nonetheless, we highlighted some relevant differences in the item loadings on each factor. Item 11 (“I have too often taken advice from the wrong people”) cross-loaded too highly between two factors and was thus removed from our analyses, following Howard [42]. Moreover, in Campbell and colleagues’ study [1], item 15 (“In the past, I have misjudged who to believe and been taken advantage of”) loaded primarily onto the Credulity component, while it loaded onto Mistrust in the Italian sample. Finally, in our sample the three factors accounted for a lesser part of the total variance than in the UK study.

The differences observed seem mainly due to linguistic and cultural factors that need further investigation. Moreover, even though referring to the presence of an EC stance, both item 11 and 15 are formulated referring to the past. After having trusted unreliable information coming from others, subjects might have shifted to a wary, distrustful attitude (EM). Further studies are needed to investigate whether and how individuals shift between the three different epistemic stances identified so far–as well as what factors mediate such changes (e.g., beliefs, life experiences, psychotherapy). However, the fit measures of the CFA corroborate the validity of a three-factor model [1]. Unexpectedly, no associations were found between ET, EM and EC, in contrast to previous results of the original validation of ETMCQ (1). These results suggest that this questionnaire needs further investigation to be able to confirm or disconfirm this theoretical model.

For what it concerns our second aim (i.e., test the mean differences in ET, EM and EC levels between males and females, as well as the correlations between the three different epistemic stances and sociodemographic characteristics such age and educational level), no differences were found between males and females, suggesting that gender has no role in accepting socially transmitted knowledge. Regarding age and the level of education, only EM showed a significant (albeit small) negative correlation with both. The negative correlation between mistrust and age appears noteworthy if combined with the result of studies showing that subjective wellbeing increase as one gets older [43], since the ability to appropriately trust others is related to lower levels of psychological symptoms. Similarly, the negative correlation between EM and level of education suggests that schooling and learning promote an adaptive ability to trust others, corroborating the results of other studies [44–46]. However, our results show the presence of some cultural differences even in the case of age and gender. In the UK sample, ET and EC were negatively associated with age, while in our sample no association was found. Moreover, EC was negatively associated with level of education, while in our sample the only epistemic stance that showed a (negative) correlation with this variable was EM. We also did not find any significant influence of gender on the three epistemic dimensions, while Campbell and colleagues [1] found that females tend to have higher scores on ET and EC, and males tend to have higher scores on EM.

With respect to the third aim (i.e., to investigate the associations between ET, EM, and EC and other aspects of psychological functioning, as well as with the presence of adverse childhood experiences), the ETMCQ showed good psychometric properties. The hypothesis that the three epistemic stances constitute independent factors was also supported by their significant correlations with distinct psychological dimensions. The three ETMCQ factors demonstrated good convergent validity as they correlated with the CTQ, RFQ, BSI, ECR-R, and B-MAS subscales. These correlations, especially the ones with the EM factor, corroborate previous theoretical contributions concerning the role of epistemic disruptions in maladaptive psychological functioning [12, 13].

ET showed small but significant positive associations with mentalized affectivity––more specifically, with the ability to identify emotions and express them. In his theory regarding mentalized affectivity (MA), Jurist [32, 47, 48] has proposed that mentalization, epistemic trust, and affect regulation are closely interconnected. Previous studies have highlighted that MA is related to the ability to use interpersonal skills and to higher levels of cognitive empathy, i.e., the ability to acknowledge affective states, epistemic states, and desire-based states [33, 49]. Moreover, according to Jurist [32], mentalized affectivity and epistemic trust remind us of the importance of the therapeutic relationship in making interventions effective. The promotion of adequate levels of ET and MA (and mentalization abilities in general) can be considered both a goal and a task of psychotherapy. Jurist argued that fostering these abilities in patients means promoting a cooperative stance within the therapeutic relationship––a factor associated with effective interventions *per se* [50, 51]––, so that patients can be open to “embrace a deeper exploration of the meaning of emotional experience in the context of their life, history, and environment” [32] and therefore develop more adaptive interpersonal schemata, beliefs, and self-conceptions. To our knowledge, there are no studies investigating the relationship between ET and MA. Our results are the first corroborating the hypothesis that an adequate ability to acquire information may be involved in emotional processes, precisely in the possibility of identifying different emotions and expressing them adaptively. This may be due to the relevance of ET in learning different abilities from others. Moreover, ET shows negative correlations with childhood trauma, psychological symptomatology, and hypo-mentalization. Adequate levels of ET also significantly correlate with lower levels of relational avoidance in romantic relationships: they may therefore represent a protective factor regarding the possibility of building more stable, trusting relationships [52]. Overall, ET seems to correlate with more adaptive psychological functioning.

EM, on the other hand, shows negative association with mentalized affectivity, specifically with emotion elaboration (i.e., the ability to modulate the intensity of affects) and expression, and positive associations with hypomentalization, childhood trauma, attachment avoidance and anxiety, and general psychological symptomatology. This is consistent with Luyten and colleagues’ [13] theoretical model about the role of childhood trauma in the development of psychopathology. Our results show that when individuals are suspicious of information coming from others, they also show difficulties understanding others in terms of mental states. Also, our findings corroborate the idea that early traumatic experiences are related to the individual’s perception that others cannot be trusted [13, 53]. Children who are exposed to adversity within their primary caregiving relationships may never lower their epistemic vigilance; they may avoid thinking about the intentions and mental states of others as a form of self-protection [54–56]. Such individuals may become hypervigilant, guarding themselves against new information even when it comes from reliable, trustworthy sources [57]. Additionally, high levels of EM seem to be associated with more difficulties in intimate relationships, i.e., with high levels of both attachment avoidance and anxiety. These dimensions are strongly associated with poorer dyadic trust: individuals with high levels of attachment anxiety and/or avoidance tend to access negative trust-related memories more easily, also showing a reduced capacity to adaptively cope with trust violations [52, 58, 59]. The rigidity and suspiciousness that characterizes EM may be a factor in generating vulnerability to the development of psychopathology, as theorized in previous studies [13, 26, 27, 60–63]. The disposition to perceive others as untrustworthy is also associated with lower levels of mentalized affectivity, which may, in turn, hinder the ability to modify maladaptive beliefs and interpersonal schemata––thus the effectiveness of psychotherapy interventions. Indeed, individuals with high levels of suspiciousness may be less inclined to recognize that they have to learn how to regulate and express emotions [64–66].

EC shows small but significant positive associations with hypomentalization, childhood trauma, attachment avoidance and anxiety, and general psychological symptomatology. Also, it is negatively associated with mentalized affectivity, specifically in relation to emotion elaboration. EC is conceptualized as a dysfunctional disposition to indiscriminately trust information coming from others, or a lack of self-protective epistemic vigilance. If a lack of epistemic trust could hinder therapy effectiveness, a lack of epistemic vigilance may impair patients’ ability to adopt an active role within the therapeutic relationship, passively accepting the therapist’s contributions without being able to incorporate change into their life in an autonomous, self-agent way––therefore not becoming able to regulate their emotions, as postulated by Jurist [32]. Our results support this idea. In addition, the association between EC and hypomentalization suggests that a lack of epistemic vigilance also hinders the ability to comprehend one’s and others’ intentions, beliefs, and affects. When humans cannot distinguish whether the information coming from others is reliable, they cannot fully learn how to perceive others accurately in terms of mental states, generating a risk of pretend mode or pseudomentalizing. The correlation between EC, hypomentalizing, and complex trauma seems to substantiate previous theoretical contributions. Knox [53], for example, has argued that childhood relational trauma may lead individuals to defensively inhibit mentalization––indeed, recognizing the abusive caregiver’s mental intentions could be further traumatizing. Complex trauma may also make individuals more prone to manipulation and exploitation from others, since it may leave them with a deep epistemic confusion about who can be trusted and who cannot, as well as about how to interpret the behavior of others towards them. As shown by our results, both pervasive suspiciousness (EM) and blind acceptance (EC) towards others are associated with attachment anxiety and avoidance; however, EC seems to bring particularly negative consequences to romantic relationships.

The associations between EM, EC, and the various aspects of mental functioning investigated in this study could have a significant clinical relevance. We believe that further studies are needed to investigate how these two forms of epistemic disruption could play a role in the development and maintenance of psychological disturbances or disorders, as well as to clarify through which mechanisms could be effective in producing change.

## Limitations

Although this study––one of the first empirical investigations of epistemic trust and its assessment––involves interesting findings, some limitations must be pointed out. First, differing from the original study by Campbell and colleagues [1], test-retest reliability was not analyzed. Future studies are needed to investigate the reliability of the ETMCQ at different times of measurement. Second, although we found a three-factor solution, the factorial structure of the ETMCQ in its Italian version does not perfectly mirror the one found in the original UK study, showing lower structural stability. The difference observed might derive from cultural differences regarding suspiciousness and credulity between the two populations, since culture is deeply involved in epistemic dimensions and social communications. As the present study is the first validation following the original one, and to our knowledge literature concerning the relationship between ET, EM, and EC is still lacking robust empirical findings, further investigations are needed. Third, our study used only self-reports methods and was conducted through an online survey, with most of the sample coming through the diffusion through social media channels. Although this method is becoming more common in psychological research, it may lead to the exclusion of part of the population (e.g., people who have little access to the Internet or difficulty using technology). This causes some limitations regarding the generalizability of our study; indeed, the socio-demographic characteristics of the sample showed that respondents tended to be more educated and with a higher economic status than the overall Italian population. The use of only self-report instruments makes us unable to detect the portion of common variance attributable to the use of similar methods (e.g., a person who tends to report less trust might also tend to report more emotional neglect). Future investigation could use mixed methods to reveal associations between constructs in more sophisticated ways. Moreover, we only investigated associations between ET, EM, EC, and related concepts, such as mentalization and mentalized affectivity (i.e., we only measured convergent validity). It would be interesting to further test for discriminant association with unrelated constructs (i.e., discriminant validity). Finally, although a self-report measure for the assessment of ET (and its disruption) represents a valuable tool for investigating this dimension both in the clinical and research field in a time-effective way, the complexity of this construct is not fully explicable via a questionnaire. Besides the use of experimental procedures of assessment, such as the one developed by Schröder-Pfeifer and colleagues [21], we believe that it would be particularly valuable to develop a structured interview o capture the complex nature of this concept.

## Conclusions

Consistently with the findings of Campbell and colleagues [1], the results of our study show that ETMCQ represents a valid and promising tool. We found an adequate replication of the original three-factor solution in the Italian population, as well as good psychometric properties.

It is our opinion that an easily handled tool such as the ETMCQ––the first self-report instrument developed for the assessment of epistemic trust, mistrust, and credulity––can be readily used in many fields: not only in the clinical arena, but also in explaining some of today’s socio-cultural dynamics. Recent studies focused on the correlations between mistrust and credulity, related constructs (including attachment avoidance and anxiety), and suspicion-based tendencies such as vaccine hesitancy [67, 68], conspiracy theories and beliefs in fake news [69–73], and other alterations in the cultural transmission of knowledge as ageism [74]. For what it concerns psychotherapy research, literature has highlighted, mainly on a theoretical level, the link between the two forms of epistemic disruption and psychopathology [2, 12, 13]. The use of an instrument such as the ETMCQ could substantiate the theoretical models concerning the role of ET in psychopathology and the psychotherapy process. Specifically, the relational nature of this concept and its involvement in therapeutic processes could enhance our understanding of the development of the therapeutic alliance. Moreover, since ET represents a transdiagnostic and transtheoretical construct, the ETMCQ could be used in further investigations on the effectiveness of different psychotherapeutic interventions. On the other hand, as previously noted, the ETMCQ could also be extremely useful in research aiming to investigate cultural and socio-political phenomena from a psychological and psychodynamic perspective, understanding the role of trust inside and outside the therapeutic room.

## Supporting information

S1 File ETMCQ items. (PDF)

S2 File Study 1 dataset. (SAV)

S3 File Study 2 dataset. (SAV)

## References

[1] Campbell C, Tanzer M, Saunders R, Booker T, Allison E, Li E, et al. Development and validation of a self-report measure of epistemic trust. PLOS ONE. 2021 Apr 16;16(4):e0250264. doi: 10.1371/journal.pone.0250264 33861805 PMC8051785

[2] Bo S, Sharp C, Fonagy P, Kongerslev M. Hypermentalizing, attachment, and epistemic trust in adolescent BPD: Clinical illustrations. Personality Disorders: Theory, Research, and Treatment. 2017;8(2):172–82. doi: 10.1037/per0000161 26691672

[3] Fonagy P, Allison E. The role of mentalizing and epistemic trust in the therapeutic relationship. Psychotherapy. 2014;51(3):372–80. doi: 10.1037/a0036505 24773092

[4] Hu L, Liu R, Zhang W, Zhang T. The Effects of Epistemic Trust and Social Trust on Public Acceptance of Genetically Modified Food: An Empirical Study from China. International Journal of Environmental Research and Public Health. 2020 Jan;17(20):7700. doi: 10.3390/ijerph17207700 33096931 PMC7593935

[5] McCraw BW. The nature of epistemic trust. Social epistemology. 2015;29(4):413–30.

[6] Orme W, Bowersox L, Vanwoerden S, Fonagy P, Sharp C. The relation between epistemic trust and borderline pathology in an adolescent inpatient sample. bord personal disord emot dysregul. 2019 Aug 28;6(1):13. 10.1186/s40479-019-0110-7 PMC6712815 31485332

[7] Talia A, Taubner S, Miller-Bottome M. Advances in research on attachment-related psychotherapy processes: seven teaching points for trainees and supervisors. Res Psychother. 2019 Dec 19;22(3):405. doi: 10.4081/ripppo.2019.405 32913812 PMC7451314

[8] Origgi G. Epistemic Injustice and Epistemic Trust. Social Epistemology. 2012 Apr 1;26(2):221–35.

[9] Fricker M. Epistemic injustice: Power and the ethics of knowing. Oxford University Press; 2007.

[10] Beck U, Giddens A, Lash S. Reflexive modernization: Politics, tradition and aesthetics in the modern social order. Stanford University Press; 1994.

[11] Giddens A. The Consequences of Modernity. Stanford: Stanford University Press; 1990. 188 p.

[12] Fonagy P, Luyten P, Allison E. Epistemic Petrification and the Restoration of Epistemic Trust: A New Conceptualization of Borderline Personality Disorder and Its Psychosocial Treatment. J Pers Disord. 2015 Oct;29(5):575–609. doi: 10.1521/pedi.2015.29.5.575 26393477

[13] Luyten P, Campbell C, Allison E, Fonagy P. The Mentalizing Approach to Psychopathology: State of the Art and Future Directions. Annu Rev Clin Psychol. 2020 May 7;16(1):297–325. doi: 10.1146/annurev-clinpsy-071919-015355 32023093

[14] Csibra G, Gergely G. Natural pedagogy. Trends in cognitive sciences. 2009;13(4):148–53. doi: 10.1016/j.tics.2009.01.005 19285912

[15] Sperber D, Clément F, Heintz C, Mascaro O, Mercier H, Origgi G, et al. Epistemic vigilance. Mind & language. 2010;25(4):359–93.

[16] Bowlby J. The bowlby-ainsworth attachment theory. Behavioral and Brain Sciences. 1979;2(4):637–8.

[17] Fonagy P, Gergely G, Target M. The parent–infant dyad and the construction of the subjective self. Journal of child psychology and psychiatry. 2007;48(3‐4):288–328. doi: 10.1111/j.1469-7610.2007.01727.x 17355400

[18] Bauer A, Stevens M, Purtscheller D, Knapp M, Fonagy P, Evans-Lacko S, et al. Mobilising social support to improve mental health for children and adolescents: A systematic review using principles of realist synthesis. PLoS One. 2021;16(5):e0251750. doi: 10.1371/journal.pone.0251750 34015021 PMC8136658

[19] Fonagy P, Luyten P, Allison E, Campbell C. Mentalizing, Epistemic Trust and the Phenomenology of Psychotherapy. PSP. 2019;52(2):94–103. doi: 10.1159/000501526 31362289

[20] Schröder-Pfeifer P, Talia A, Volkert J, Taubner S. Developing an assessment of epistemic trust: a research protocol. Research in Psychotherapy: Psychopathology, Process and Outcome [Internet]. 2018 Dec 18 [cited 2022 Nov 2];21(3). Available from: https://www.researchinpsychotherapy.org/index.php/rpsy/article/view/330 10.4081/ripppo.2018.330 PMC7451362 32913771

[21] Schröder-Pfeifer P, Georg AK, Talia A, Volkert J, Ditzen B, Taubner S. The Epistemic Trust Assessment—An experimental measure of epistemic trust. Psychoanalytic Psychology. 2022 Jan;39(1):50–8.

[22] Grice P. Studies in the Way of Words. Harvard University Press; 1989.

[23] Sperber D, Wilson D. Relevance: Communication and cognition. Vol. 142. Citeseer; 1986.

[24] Wilson D, Sperber D. Relevance theory. The handbook of pragmatics. Edited by L Horn G Ward Oxford, Blackwell. 2004;607–32.

[25] De Carli P, Tagini A, Sarracino D, Santona A, Bonalda V, Cesari PE, et al. Like grandparents, like parents: Empirical evidence and psychoanalytic thinking on the transmission of parenting styles. Bull Menninger Clin. 2018;82(1):46–70. doi: 10.1521/bumc_2017_81_11 29120666

[26] Fonagy P, Luyten P, Allison E, Campbell C. What we have changed our minds about: Part 2. Borderline personality disorder, epistemic trust and the developmental significance of social communication. 2017;4(1):1–12. doi: 10.1186/s40479-017-0062-8 28405338 PMC5387344

[27] Knapen S, Hutsebaut J, van Diemen R, Beekman A. Epistemic trust as a psycho-marker for outcome in psychosocial interventions. Journal of Infant, Child, and Adolescent Psychotherapy. 2020;19(4):417–26.

[28] Fonagy P, Luyten P, Moulton-Perkins A, Lee YW, Warren F, Howard S, et al. Development and validation of a self-report measure of mentalizing: The reflective functioning questionnaire. PloS one. 2016;11(7):e0158678. doi: 10.1371/journal.pone.0158678 27392018 PMC4938585

[29] Woźniak-Prus M, Gambin M, Cudo A, Sharp C. Investigation of the Factor Structure of the Reflective Functioning Questionnaire (RFQ-8): One or Two Dimensions? Journal of Personality Assessment. 2021;1–11. 10.1080/00223891.2021.2014505 35015610

[30] Morandotti N, Brondino N, Merelli A, Boldrini A, De Vidovich GZ, Ricciardo S, et al. The Italian version of the Reflective Functioning Questionnaire: Validity data for adults and its association with severity of borderline personality disorder. PLoS One. 2018;13(11):e0206433. doi: 10.1371/journal.pone.0206433 30383803 PMC6211684

[31] Greenberg DM, Rudenstine S, Alaluf R, Jurist EL. Development and validation of the Brief‐Mentalized Affectivity Scale: Evidence from cross‐sectional online data and an urban community‐based mental health clinic. Journal of Clinical Psychology. 2021;77(11):2638–52. doi: 10.1002/jclp.23203 34260738

[32] Jurist EL. Minding emotions: cultivating mentalization in psychotherapy. New York: The Guilford Press; 2018. 200 p. (Psychoanalysis and psychological science).

[33] Liotti M, Spitoni GF, Lingiardi V, Marchetti A, Speranza AM, Valle A, et al. Mentalized affectivity in a nutshell: Validation of the Italian version of the Brief-Mentalized Affectivity Scale (B-MAS). Plos one. 2021;16(12):e0260678. doi: 10.1371/journal.pone.0260678 34855839 PMC8639076

[34] Bernstein DP, Stein JA, Newcomb MD, Walker E, Pogge D, Ahluvalia T, et al. Development and validation of a brief screening version of the Childhood Trauma Questionnaire. Child Abuse & Neglect. 2003 Feb 1;27(2):169–90. doi: 10.1016/s0145-2134(02)00541-0 12615092

[35] Grassi-Oliveira R, Stein LM. Childhood maltreatment associated with PTSD and emotional distress in low-income adults: The burden of neglect. Child Abuse & Neglect. 2008 Dec;32(12):1089–94. doi: 10.1016/j.chiabu.2008.05.008 19036445

[36] Sacchi C, Vieno A, Simonelli A. Italian validation of the Childhood Trauma Questionnaire—Short Form on a college group. Psychological Trauma: Theory, Research, Practice, and Policy. 2018;10(5):563–71. doi: 10.1037/tra0000333 29016156

[37] Fraley RC, Waller NG, Brennan KA. An item response theory analysis of self-report measures of adult attachment. Journal of Personality and Social Psychology. 2000;78(2):350–65. doi: 10.1037//0022-3514.78.2.350 10707340

[38] Busonera A, Martini PS, Zavattini GC, Santona A. Psychometric properties of an Italian version of the Experiences in Close Relationships-Revised (ECR-R) Scale. Psychological reports. 2014;114(3):785–801. doi: 10.2466/03.21.PR0.114k23w9 25074302

[39] Derogatis LR. The brief symptom inventory (BSI): administration, scoring & procedures manual-II. Clinical Psychometric Research; 1992.

[40] Kline RB. Principles and practice of structural equation modeling. Guilford publications; 2015.

[41] Mascaro O, Sperber D. The moral, epistemic, and mindreading components of children’s vigilance towards deception. Cognition. 2009;112(3):367–80. doi: 10.1016/j.cognition.2009.05.012 19540473

[42] Howard MC. A review of exploratory factor analysis decisions and overview of current practices: What we are doing and how can we improve? International Journal of Human-Computer Interaction. 2016;32(1):51–62.

[43] Steptoe A, Deaton A, Stone AA. Subjective wellbeing, health, and ageing. The Lancet. 2015 Feb 14;385(9968):640–8. doi: 10.1016/S0140-6736(13)61489-0 25468152 PMC4339610

[44] Hooghe M, Marien S, de Vroome T. The cognitive basis of trust. The relation between education, cognitive ability, and generalized and political trust. Intelligence. 2012 Nov 1;40(6):604–13.

[45] Nannestad P. What have we learned about generalized trust, if anything? Annu Rev Polit Sci. 2008;11:413–36.

[46] Borgonovi F. The relationship between education and levels of trust and tolerance in Europe1. The British Journal of Sociology. 2012;63(1):146–67. 22404393 10.1111/j.1468-4446.2011.01397.x

[47] Jurist EL. Mentalized affectivity. Psychoanalytic Psychology. 2005;22(3):426.

[48] Jurist EL. Minds and yours: New directions for mentalization theory. In: Mind to mind: Infant research, neuroscience, and psychoanalysis. New York, NY, US: Other Press; 2008. p. 88–114.

[49] Greenberg DM, Kolasi J, Hegsted CP, Berkowitz Y, Jurist EL. Mentalized affectivity: A new model and assessment of emotion regulation. PLOS ONE. 2017 Oct 18;12(10):e0185264. doi: 10.1371/journal.pone.0185264 29045403 PMC5646776

[50] Monticelli F, Liotti M. Motivational Monitoring: How to Identify Ruptures and Impasses and Enhance Interpersonal Attunement. J Contemp Psychother. 2021 Jun 1;51(2):97–108.

[51] Monticelli F, Tombolini L, Guerra F, Liotti M, Monticelli C, Gasperini E, et al. Using Motivational Monitoring to Evaluate the Efficacy of Self-disclosure and Self-involving Interventions. Journal of Contemporary Psychotherapy [Internet]. 2022 Feb 28; Available from: 10.1007/s10879-022-09533-y

[52] Campbell L, Stanton SC. Adult attachment and trust in romantic relationships. Current Opinion in Psychology. 2019;25:148–51. doi: 10.1016/j.copsyc.2018.08.004 30096516

[53] Knox J. Epistemic Mistrust: A Crucial Aspect of Mentalization in People with a History of Abuse?: Epistemic Mistrust. British Journal of Psychotherapy. 2016 May;32(2):226–36.

[54] Liotti G, Liotti M. Reflections on some contributions to contemporary psychotraumatology in the light of Janet’s critique of Freud’s theories 1. In: Rediscovering Pierre Janet. Routledge; 2019.

[55] Attachment Fonagy P. and borderline personality disorder. Journal of the american psychoanalytic association. 2000;48(4):1129–46. 11212185 10.1177/00030651000480040701

[56] Oehlman Forbes D, Lee M, Lakeman R. The role of mentalization in child psychotherapy, interpersonal trauma, and recovery: A scoping review. Psychotherapy. 2021;58(1). doi: 10.1037/pst0000341 32881544

[57] Luyten P, Fonagy P. Mentalizing and trauma. Handbook of mentalizing in mental health practice. 2019;2:79–99.

[58] Fitzpatrick J, Lafontaine MF. Attachment, trust, and satisfaction in relationships: Investigating actor, partner, and mediating effects. Personal Relationships. 2017;24(3):640–62.

[59] Mikulincer M, Florian V. The relationship between adult attachment styles and emotional and cognitive reactions to stressful events. Attachment theory and close relationships. 1998;143–65.

[60] Boldrini T, Girardi P, Clerici M, Conca A, Creati C, Di Cicilia G, et al. Consequences of the COVID-19 pandemic on admissions to general hospital psychiatric wards in Italy: Reduced psychiatric hospitalizations and increased suicidality. Prog Neuropsychopharmacol Biol Psychiatry. 2021 Aug 30;110:110304. doi: 10.1016/j.pnpbp.2021.110304 33737215 PMC8569419

[61] Kamphuis JH, Finn SE. Therapeutic assessment in personality disorders: Toward the restoration of epistemic trust. Journal of Personality Assessment. 2018; doi: 10.1080/00223891.2018.1476360 29873526

[62] Li ET, Midgley N, Luyten P, Sprecher EA, Campbell C. Mapping the journey from epistemic mistrust in depressed adolescents receiving psychotherapy. Journal of Counseling Psychology. 2022;No Pagination Specified-No Pagination Specified. doi: 10.1037/cou0000625 35737539

[63] Santona A, Milesi A, Tognasso G, Gorla L, Parolin L. Anxiety in Attachment and Sexual Relationships in Adolescence: A Moderated Mediation Model. International Journal of Environmental Research and Public Health. 2022 Jan;19(7):4181. doi: 10.3390/ijerph19074181 35409864 PMC8998572

[64] Maffei C, Fossati A, Lingiardi V, Madeddu F, Borellini C, Petrachi M. Personality maladjustment, defenses, and psychopathological symptoms in nonclinical subjects. Journal of Personality Disorders. 1995;9(4):330.

[65] Parolin LAL, Benzi IMA, Fanti E, Milesi A, Cipresso P, Preti E. Italia Ti Ascolto [Italy, I am listening]: an app-based group psychological intervention during the COVID-19 pandemic. Research in Psychotherapy: Psychopathology, Process, and Outcome. 2021;24(1). 10.4081/ripppo.2021.517 PMC8082536 33937116

[66] Tanzilli A, Giuseppe MD, Giovanardi G, Boldrini T, Caviglia G, Conversano C, et al. Mentalization, attachment, and defense mechanisms: a Psychodynamic Diagnostic Manual-2-oriented empirical investigation. Research in Psychotherapy: Psychopathology, Process and Outcome [Internet]. 2021 Mar 29 [cited 2022 Aug 22];24(1). Available from: https://www.researchinpsychotherapy.org/index.php/rpsy/article/view/531 doi: 10.4081/ripppo.2021.531 33937117 PMC8082535

[67] Nihlén Fahlquist J. Vaccine hesitancy and trust. Ethical aspects of risk communication. Scand J Public Health. 2018 Mar 1;46(2):182–8. doi: 10.1177/1403494817727162 28847220

[68] Tanzilli A, Cibelli A, Liotti M, Fiorentino F, Williams R, Lingiardi V. Personality, Defenses, Mentalization, and Epistemic Trust Related to Pandemic Containment Strategies and the COVID-19 Vaccine: A Sequential Mediation Model. International Journal of Environmental Research and Public Health. 2022 Jan;19(21):14290. doi: 10.3390/ijerph192114290 36361183 PMC9656964

[69] Frenken M, Imhoff R. Don’t trust anybody: Conspiracy mentality and the detection of facial trustworthiness cues. Applied Cognitive Psychology [Internet]. [cited 2022 Jul 12];n/a(n/a). Available from: https://onlinelibrary.wiley.com/doi/abs/10.1002/acp.3955

[70] Green R, Douglas KM. Anxious attachment and belief in conspiracy theories. Personality and Individual Differences. 2018;125:30–7.

[71] Leone L, Giacomantonio M, Williams R, Michetti D. Avoidant attachment style and conspiracy ideation. Personality and Individual Differences. 2018 Nov 1;134:329–36.

[72] Pierre JM. Mistrust and Misinformation: A Two-Component, Socio-Epistemic Model of Belief in Conspiracy Theories. Journal of Social and Political Psychology. 2020 Oct 12;8(2):617–41.

[73] Tanzer M, Campbell C, Saunders R, Luyten P, Booker T, Fonagy P. Acquiring knowledge: Epistemic trust in the age of fake news [Internet]. PsyArXiv; 2021 [cited 2022 Aug 3]. Available from: https://psyarxiv.com/g2b6k/

[74] Henry RS, Perrin PB, Smith ER. The Underpinnings of Ageism: Multiple Mediational Model of Epistemological Style, Social Dominance Orientation, Right-Wing Authoritarianism, and Ageist Attitudes. Ferraro FR, editor. Journal of Aging Research. 2019 Nov 3;2019:3672725. 31781393 10.1155/2019/3672725 PMC6874979

