# Supplementary material for: Unpacking trust: The Italian validation of the Epistemic Trust, Mistrust, and Credulity Questionnaire (ETMCQ)
Source: PLoS One. 2023 Jan 26;18(1):e0280328. doi: 10.1371/journal.pone.0280328 (PMC9879475; doi:10.1371/journal.pone.0280328)
Supplement: S1 File — (PDF) [file pone.0280328.s001.pdf]

	Non vero	Molto vero						
1. Quando ho un problema personale di solito chiedo consiglio agli altri	1	2	3	4	5	6	7	
2. Trovo più facile credere alle informazioni che provengono da chi mi conosce bene	1	2	3	4	5	6	7	
3. Preferisco andare a cercarmi da solo le cose su internet piuttosto che chiedere informazioni alle altre persone	1	2	3	4	5	6	7	
4. Spesso mi sembra che gli altri non capiscano cosa voglio o di cosa ho bisogno	1	2	3	4	5	6	7	
5. Vengo spesso considerato ingenuo/a perché credo a quasi tutto quello che la gente mi dice	1	2	3	4	5	6	7	
6. Quando parlo con varie persone mi faccio convincere da ciò che dicono, anche se prima credevo cose diverse	1	2	3	4	5	6	7	
7. A volte parlare con persone che mi conoscono da tanto tempo mi aiuta ad acquisire un punto di vista diverso su di me	1	2	3	4	5	6	7	
8. Trovo utile imparare da quello che gli altri mi raccontano a proposito delle loro esperienze	1	2	3	4	5	6	7	
9. Se ti fidi troppo di quello che la gente ti dice, finisci per rimanere ferito	1	2	3	4	5	6	7	
10. Quando qualcuno mi dice qualcosa la mia prima reazione è chiedermi perché me la sta dicendo	1	2	3	4	5	6	7	
11. Mi è capitato troppo spesso di seguire i consigli delle persone sbagliate	1	2	3	4	5	6	7	
12. Le persone mi dicono che sono troppo facilmente influenzabile	1	2	3	4	5	6	7	
13. Se non so cosa fare, il mio primo impulso è chiedere a qualcuno della cui opinione mi fido	1	2	3	4	5	6	7	
14. Di solito non metto in pratica i consigli degli altri, anche quando penso che probabilmente sono validi	1	2	3	4	5	6	7	
15. In passato, ho creduto alle persone sbagliate e gli altri si sono approfittati di me.	1	2	3	4	5	6	7	

This was the original version administered to participants.
Note: Item 11 was excluded from the scoring.
